# Clinical Trial for Cartilage Conduction Hearing Aid in Indonesia

**DOI:** 10.3390/audiolres11030038

**Published:** 2021-08-13

**Authors:** Ronny Suwento, Dini Widiarni Widodo, Tri Juda Airlangga, Widayat Alviandi, Keisuke Watanuki, Naoko Nakanowatari, Hiroshi Hosoi, Tadashi Nishimura

**Affiliations:** 1 Department of Otorhinolaryngology Head and Neck Surgery, Cipto Mangunkusumo Hospital—Faculty of Medicine, Universitas Indonesia, Jakarta 10430, Indonesia; airlanggamd@gmail.com (T.J.A.); widayat_alviandi@yahoo.com (W.A.); 2 RION Co., Tokyo 185-8533, Japan; watanuki@rion.co.jp (K.W.); naoko@rion.co.jp (N.N.); 3 Nara Medical University, Kashihara 634-8522, Nara, Japan; hosoi@naramed-u.ac.jp (H.H.); t-nishim@naramed-u.ac.jp (T.N.)

**Keywords:** cartilage conduction hearing aid, microtia, hearing function, clinical trial

## Abstract

Hearing improvement represents one of the may valuable outcomes in microtia and aural atresia reconstruction surgery. Most patients with poor development in their hearing function have had a severe microtia. Conventional methods to improve hearing function are bone conduction and bone anchored hearing aids. Cartilage conduction hearing aids (CCHA) represents a new amplification method. This study assessed the outcomes and evaluated the impact and its safety in the patients with microtia and aural atresia whose hearing dysfunction did not improve after surgery for ear reconstruction in our hospital. Hearing functions were evaluated with pure tone audiometry or sound field testing by behavioral audiometry and speech audiometry before and after CCHA fitting. As a result, there was a significant difference between unaided and aided thresholds (*p* < 0.001). Speech recognition threshold and speech discrimination level also significantly improved with CCHA. The average functional gains of 14 ears were 26.9 ± 2.3 dB. Almost all parents of the patients reported satisfaction with the performance of CCHA, and daily communication in children with hearing loss also became better than usual.

## 1. Introduction

Microtia is a congenital auricular malformation that usually occurs in conjunction with ear canal atresia, and ranges from mild structural abnormalities to the complete absence of the ear (anotia). It can occur unilaterally or bilaterally. In unilateral cases, the right side is more affected. The prevalence rate of microtia ranges from 0.83 to 17.4 per 10,000 [1]. In the ENT outpatient clinic, Dr. Cipto Mangunkusumo National Hospital, Jakarta, there were 207 microtia ears in 2008–2014, and 173 ears underwent surgery in the patients aged from 6 to 12 years of age. Between 2017 and 2018, there were 32 new microtia cases, aged between 1 month and 14 years. Male babies have been more frequently affected than female babies (2:1).

Microtia patients have three main problems, namely functional, aesthetic, and psychosocial problems [1]. Microtia surgery has been proved to lower psychological stressors which may impact the mental development of children with microtia [2]. Hearing habilitation for infants and children with microtia who are still in developing age should be performed without waiting for reconstructive surgery. One of the options for microtia hearing habilitation that is commonly used in Indonesia is the installation of bone conduction hearing aid. However, this method is ineffective due to several obstacles such as the difficulty in obtaining the correct hearing aid input, transducer pressure (which makes it unstable on bones), the occurrence of skin laceration due to transducer pressure, and its higher cost than air conductive hearing aids. Another option is the installation of a bone anchored hearing aid (BAHA), which is even more expensive and requires surgery. In addition, complications can also occur after the BAHA implantation surgery [3,4,5].

When children become old enough to undergo ear reconstruction surgery, hearing habilitation can be assisted by performing atresiaplasty. Hearing improvement becomes one of valuable outcomes of microtia reconstruction surgery [2]. About 64% patients gain significant hearing improvement after atresiaplasty. This result remained stable for up to three years post-surgery. Most patients who did not develop their hearing function had a severe degree of microtia. Severe malformed middle ear and stenosis of the ear canal are associated with a negative impact on auditory development [2,6]. However, atresiaplasty is also often a dilemma since it can result in restenosis of the ear canal. Ear canal restenosis is caused by circumference wounds (360°) that can cause contractures as well as fibrosis of the soft tissue around the wound. These factors cause the amplification effort to be hampered so that the patient still has hearing problems after the reconstruction procedure [7].

Hearing amplification technology development is needed to overcome these obstacles, especially for infants and children. Cartilage conduction hearing aid (CCHA) developed by Hosoi and colleagues provides new hope and can be an alternative option for overcoming hearing amplification problems in microtia cases [8,9,10,11,12]. Cartilage conduction is a newly suggested transduction form whose characteristics are different from air and bone conductions. [13,14,15,16,17,18,19,20] CCHA has several advantages such as sound clarity, sound localization, and more stable connection between the transducer and the cartilage surface [20,21,22]. The transducer can be attached to the cartilage part in humans via a special double-sided tape. As previous studies stated [12,23,24,25,26,27], CCHA can be used for sound transmission even in aural atresia. Most issues with bone conduction (BC) hearing aids are related to the properties of the transducer and the form of conduction. For cartilage conduction (CC), the transducer is designed to vibrate the aural cartilage rather than the skull bone; therefore, it is small and lightweight. By inserting the transducer into the cavity of the concha, a headband is not needed for fixation. It is held in place by the combination of its own weight and the stiffness of the concha cartilage. In addition to its cosmetic advantages, the fixation of a CC transducer is more comfortable and convenient than that of a BC transducer [12]. This alternative conduction method may solve the issues related to BC hearing aids.

The purpose of this clinical trial is to find out whether CCHA is useful for patients with severe conductive hearing loss due to aural atresia. This study also aimed to assess the outcomes and to evaluate the impact and safety of CCHA in the patients with microtia and aural atresia whose hearing dysfunction were difficult to improve by following ear surgery reconstruction at the ENT Department, Dr. Cipto Mangunkusumo National Hospital, Jakarta.

## 2. Method

### 2.1. Participants

This is a quasi-experimental study comparing outcomes before and after intervention. We used purposive sampling to choose subjects based on inclusion criteria. This clinical trial was conducted at the ENT Department, Dr Cipto Mangunkusumo National Hospital—Faculty of Medicine Universitas Indonesia, Jakarta from August 2019 to January 2020. This study has been approved by the Ethics Committee of Faculty of Medicine, Universitas Indonesia with an official letter of 17 June 2019, Number KET/UN2/ETIK/PPM.00.02/2019 with Protocol number: 19-05-0533. We clarify cases which were suitable to our inclusion criteria. Subjects with sensorineural hearing loss were excluded. Subjects’ parents were provided written informed consent after being informed the nature of the procedure and purpose of this study.

The present study evaluated 10 children diagnosed as microtia and aural atresia. Six subjects (60%) were female and four were males (40%). Eight subjects had bilateral microtia and aural atresia while the other two subjects were only one ear (unilateral). The total number involved 18 ears (8 right ears and 10 left ears) of microtia and aural atresia. Subjects’ average age was 12.4 ± 3.1 years; the youngest was 9 years while the oldest was 19 years. All subjects were classified as microtia grade III, with a Jahrsdoerfer score less than 7. All subjects had undergone auricular and aural atresia reconstruction surgery and did not get hearing improvement after surgery. Only one patient (number 10) had ever used conventional bone conduction hearing aid bilaterally, but it had been used for only four months because of discomfort. In patient nine, Bone anchored hearing aid (BAHA) was installed before but was released due to complications (excessive granulation tissue).

Initially, we filled in the complete identity of the subjects in the research form, interviewed the parents, and reviewed the subjects’ medical records. This was followed by air and bone conduction pure tone audiometry or behavioral audiometry or bone conduction ABR, and speech audiometry. Clinical audiometer (AC 40; Interacoustics, Middelfart, Denmark) and ABR Bio-Logic Navigator Pro (Natus Medical Inc., San Carlos, CA, USA) were used for the measurement. Speech audiometry was performed using Otometrics Madsen Astera (Natus, Taastrup, Denmark). In the initial audiology test, only seven patients completed pure tone audiometry, with average hearing threshold taken from 500, 1000, 2000 and 4000 Hz. The other three patients were not cooperative to have pure tone audiometry performed; subjective auditory responses were evaluated with behavioral audiometry in one of them and the audiology response of the other two patients were determined based on the tone burst (TB) bone conduction ABR.

Based on the results of the initial audiological tests, the diagnosis of hearing loss in 18 ears was conductive hearing loss with a degree of profound hearing loss in 2 ears, severe hearing loss in 12 ears, and 4 moderate hearing loss (Table 1).

### 2.2. CCHA Fitting and Evaluations 

**Figure 1:**
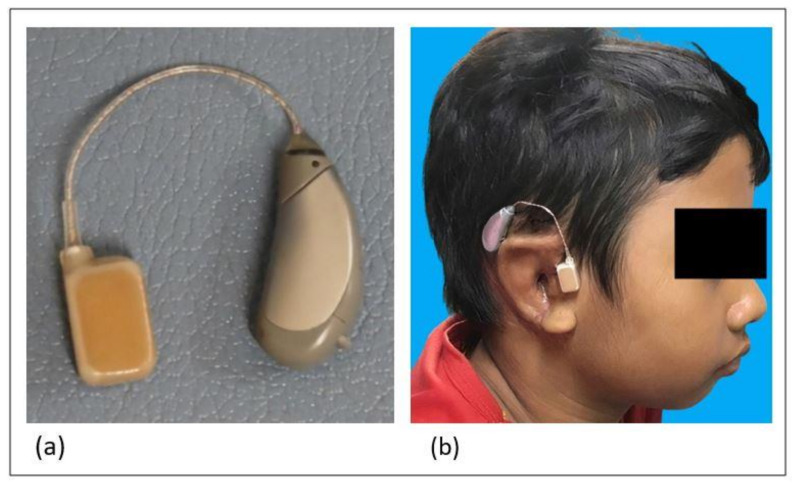
Cartilage conduction hearing aid (**a**) and the appearance of it on a patient ear (**b**).

HB-J1CC CCHA (Rion Co. Ltd., Tokyo, Japan) was used for CCHA fitting (Figure 1a). An ear impression was taken to prepare the CCHA transducer when the ear-chip type transducer was necessary. CCHAs with ear-chip type transducer were fitted in patient three (left ear) and five (both ears), while the simple type transducer was affixed to the external ear cartilage with a double-sided skin tape (#1522; 3M Japan Limited, Tokyo, Japan) in others (Figure 1b). The transducers were placed on the tragal area which consist mostly of cartilage. CCHA adjustments was performed based on functional gains. Unaided and aided thresholds were measured in the same day by sound field test, and functional gains were obtained.

Speech audiometry assessments were also performed by calculating speech recognition threshold (SRT) and speech discrimination score (SDS). The intensity level at which the patient could correctly repeat 50% of spondee words (single words which comprise two syllables with equal emphasis placed on each syllable) was measured and defined as SRT. The SRTs should correspond roughly to the average pure tone audiometry thresholds at 500, 1000 and 2000 Hz. Meanwhile, The SDS (also called word recognition score) is a score of the number of words correctly repeated, expressed as a percentage of correct (discrimination score) or incorrect (discrimination loss).

Furthermore, speech discrimination level (SDL) was defined as the lowest level at which enough SDS was obtained for communication. SDL indicates the patient’s ability to hear and understand speech at typical conversation levels, which helps us to predict the potential benefits from the amplification.

The final session incorporated with sound field testing (unaided and aided) and the subjective benefits of CCHA use in a daily life were evaluated with a questionnaire for parents. 

## 3. Results

Hearing threshold improvements which are assessed based on functional gain were performed in 14 ears. The functional gains were obtained as the result of the difference value between aided and unaided audiometric behavioral threshold. Patient one, four, and five were difficult to perform hearing and speech examination and CCHA fitting on. It should be explained that the results of behavioral audiometry in patient one are inconsistent during the three months of the examination sessions. The patient’s emotions during examination sessions were unstable. The same condition also happened to patients four and five, who were not cooperative. However, at the last session, the behavioral audiometry was successfully conducted in patient four, as the results were reliable. Patient five could not produce any reliable result because of their uncooperativeness. Thus, we fitted their CCHA based on their previous bone conduction ABR result. Nonetheless, patients one, four, and five were subjectively seen more comfortable and wanted to wear CCHA. 

Functional gains could be obtained in 14 ears of 8 patients. Obtained values ranged from 11.25 dB to 46.25 dB (Table 2). The average functional gain of 14 ears was 26.9 ± 2.3 dB. The greatest improvements in hearing threshold among the bilateral fitting cases were 35 and 38.75 dB in patient four. For unilateral fitting cases, the largest functional gain was 46.25 dB observed in the left ear of patient nine.

A statistical test was performed to evaluate the outcome significance. Firstly, a Shapiro–Wilk test was conducted to evaluate data normality for subjects with less than 50 samples. As the P value is higher than 0.05, data distribution was normal and mean and standard deviation were used to present the data. Secondly, parametric statistical test was performed. Based on the paired *T*-test, mean difference of functional gain obtained was 26.9 ± 2.3 dB (95% CI). It was concluded that there is a statistically significant difference (*p* < 0.001) in the average hearing threshold between unaided and aided conditions. (Table 3).

SRT results were successfully obtained in 12 ears of 7 patients. SRT results was improved in all seven patients, with improvements ranged from 24 to 64 dB (Table 2). The average SRT improvement was 36.4 ± 12.6 dB. Improvement of SDL values also occurred in all seven subjects with median value of 40 dB and minimum and maximal values of 20 dB and 40 dB, respectively (Table 2). The smallest aided SDL value recorded was 50 dB. The SDL improvement in patients with CCHA might be larger than the obtained values. 

Almost all subjects’ parents reported satisfaction with the performance of CCHA; subject daily communication becomes better, and it was reported that the subjects felt more comfortable with CCHA installed. After six months of CCHA installation, no disturbing problems have been reported. No adverse effects or allergies were found due to double-sided tape. The results of the evaluation of hearing aid adaptability test questionnaire to subjects’ parents were as follows: good adaptability (90%); the effect of improvement was felt immediately (85%); ease operability factor (90%); device appearance (100%); and comfort of use (90%).

## 4. Discussions

Overall, the hearing threshold (functional gain), and the ability to understand speech (speech audiometry) of all subjects improved after CCHA installation, which agreed with the previous clinical trial [12]. The benefit obtained by CCHA users in this study were not the same but varied. This variable benefit might result from the individual pathology. A person might have poorer speech discrimination scores than others due to the way the cochlear hair cells or auditory nerve had been damaged. It might also be due to a patient’s personality, or a combination of other factors. 

One of the difficulties in determining the audiological status of a patient with microtia and aural atresia is a psychosocial problem that causes difficulties in performing a hearing examination. This has been conveyed in various studies including by Li et.al., who studied 170 microtia patients [28]. They reported that microtia and aural atresia patients aged 8–10 years (boy) and 11–13 years (girl) had a high incidence of social problems in the form of interpersonal sensitivity, depression, anxiety, and hostility. This also occurred in the microtia and aural atresia patients in our study, which consisted of 6 girls and 4 boys, with an age range of 9–19 years, so that only 6 patients could undergo pure tone audiometry. As an alternative procedure, behavioral audiometry was performed for 8 patients (12 ears), while 2 other patients, aged 10 and 9 years, were still unable to undergo behavioral audiometry. In these two patients, bone conduction ABR, allowing the identification of the type and the degree of conduction hearing loss, was performed to assess the cochlear integrity [29]. Due to psychosocial problems, the two subjects could not complete another audiological test session. However, the parents of both subjects still wanted to participate in the installation of the CCHA, and both subjects felt better subjectively after using CCHA. 

Evaluating the performance in the patients with difficulty in behavioral audiometry, the real ear measurement is effective in normal anatomical ears [30], and the simulator for CC is also beneficial to the estimation [31,32]. Unfortunately, the ear canal was substantially absent in the atretic ears, and these measurements cannot exactly reflect the signal transmission to the cochlea. Technological developments in objectively evaluating the performance in the atretic ears are necessary since a suitable candidate for CCHA is a patient with aural atresia. Subject parents reported that the CCHA unit was very light, relatively small in size, and that it therefore looks better cosmetically. Most of the subjects used double-sided tape for fixation of transducers and hearing aid units; only two subjects could use the ear-chip type transducer. Sound transmission is quite good with the use of double-sided tape and does not cause pressure on the skin attachment. The similar audiometric outcomes have been reported by Nishimura et al. [12]. The style of the transducer fixation using double-sided tapes and the type of aural atresia had no significant influence on the functional gains [12].

The limitation of this study is its small sample size as we only had 10 subjects who met the inclusion criteria. In addition, the subject was difficult to follow up due to the travel restriction policy during Covid-19 pandemic. Some of them also had psychosocial problem that cause difficulties in performing hearing tests. 

## 5. Conclusions

The CCHA outcome and benefits in this study were varied. This is caused by different respond to the device. This variable benefit may also result from individual pathology. Based on audiometric tests and interviews with the subject parents, CCHA is a hearing aid choice that provides optimal hearing amplification. CCHA is a suitable and profitable option of hearing rehabilitation for microtia and aural atresia patients who do not receive benefit or amplification following ear reconstruction surgery.

## Figures and Tables

**Table 1:** Characteristics of 10 Patients with Microtia and Aural Atresia.

Patient	Sex	Age (Year)	Pure Tone/Behavioral Audiometry (dB HL) [500, 1000, 2000, 4000 Hz]		TB Bone ABR (dB nHL)		Degree of Conductive Hearing Loss	
			R	L	R	L	R	L
1	F	13	107.5/ 67.5	101.3/ 68.8	-	-	Profound/ Severe	Profound/ Severe
2	M	9	NA/ 50	NA/ 50	-	-	- Moderate	- Moderate
3	F	14	66.3/ 62.5	62.5/ 53.8	-	-	Severe/ Severe	Severe/ Moderate
4	F	10	NA	NA	60	60	Severe	Severe
5	M	9	NA	NA	55	65	Moderate	Severe
6	M	12	60.0/ 51.3	61.3/ 56.3	-	-	Severe/ Moderate	Severe/ Moderate
7	F	13	70.0/ 32.5	58.8/ 28.8	-	-	Severe/ Mild	Moderate/ Mild
8	F	19	Normal/ Normal	68.8/ 70.0	-	-	Normal/ Normal	Severe/ Severe
9	F	15	Normal/ Normal	82.5/ 82.5	-	-	Normal/Normal	Severe/Severe
10	F	10	61.3/ 68.8	65.0/ 65.0	-	-	Severe/ Severe	Severe/ Severe

M, male; F, female; R, right ear; L, left ear; NA, not available; ABR, auditory brainstem response.

**Table 2:** CCHA fitting and its outcome.

Patient	Ear-Chip	DFT	Ear	Unaided/ Aided Thresholds (dB HL)	Functional Gain (dB)	Unaided/ Aided SRTs (dB HL)	SRT-I (dB)	Unaided/ Aided SDLs (dB HL)	SDL-I (dB)
1	No	Yes	R	NC/90	NC	NC	NC	NC	NC
	No	Yes	L	NC/68.8	NC	NC	NC	NC	NC
2	No	Yes	R	50/27.5	22.5	68/46	22	100/60	40
	No	Yes	L	50.0/28.8	21.3	72/47	25	100/60	40
3	No	Yes	R	52.5/31.3	21.3	93/49	44	NC/90	-
	Yes	No	L	48.8/37.5	11.3	82/60	22	100/80	20
4	No	Yes	R	66.3/31.3	35.0	NC	NC	NC	NC
	No	Yes	L	63.8/25.0	38.8	NC	NC	NC	NC
5	Yes	No	R	NC	NC	NC	NC	NC	NC
	Yes	No	L	NC	NC	NC	NC	NC	NC
6	No	Yes	R	50.0/27,5	22.5	75/30	45	?/60	-
	No	Yes	L	50.0/28.8	21.3	92/28	64	100/60	40
7	No	Yes	R	65.0/38.8	26.3	81/42	39	90/50	40
	No	Yes	L	56.3/30.0	26.3	73/37	36	80/50	30
8	-	-	R	Normal	-	-	-	-	-
	No	Yes	L	68.8/38.8	30.0	74/50	24	90/60	30
9	-	-	R	Normal	-	-	-	-	-
	No	Yes	L	76.3/30.0	46.3	85/50	35	90/60	30
10	No	Yes	R	57.5/32.5	25.0	76/28	48	90/50	40
	No	Yes	L	61.3/32.5	28.8	75/42	33	90/50	40

DFT: double-sided tape for transducer and hearing aid unit; SRT: speech recognition score; SDL: speech discrimination level; I: improvement; R: Right ear; L: Left ear; NC: not cooperative.

**Table 3:** Averaged unaided/aided thresholds and functional gain.

Unaided Threshold (n = 14)	Aided Threshold (n = 14)	Functional Gain (95% Confidence Interval)	*P* Value (Paired *T*-Test)
58.3 ± 2.3 dB HL	31.4 ± 1.1 dB HL	26.9 ± 2.3 dB	<0.001

## Data Availability

The data used to support the findings of this study are available within the article.
